# Association Between Dimensions of Professional Burnout and Turnover Intention Among Nurses Working in Hospitals During Coronavirus Disease (COVID-19) Pandemic in Iran Based on Structural Model

**DOI:** 10.3389/fpubh.2022.860264

**Published:** 2022-05-25

**Authors:** Leila Karimi, Mehdi Raei, Akram Parandeh

**Affiliations:** ^1^Department of Community Health, Behavioral Sciences Research Center, Life Style Institute, Nursing Faculty, Baqiyatallah University of Medical Sciences, Tehran, Iran; ^2^Department of Epidemiology and Biostatistics, Health Research Center, Life Style Institute, Baqiyatallah University of Medical Sciences, Tehran, Iran; ^3^Department of Community Health, Medicine, Quran and Hadith Research Center, Nursing Faculty, Baqiyatallah University of Medical Sciences, Tehran, Iran

**Keywords:** COVID-19, nurses, pandemics, professional burnout, psychological, workplace, personnel turnover

## Abstract

**Purpose:**

This study was done to assess the dimensions of professional burnout and turnover intention among nurses working in hospitals during the coronavirus disease 2019 (COVID-19) pandemic in Iran based on a structural model.

**Methods:**

This cross-sectional study was performed among 170 nurses working in two referral hospitals of COVID-19 in Tehran Province, Iran, from September to December 2020. Data were collected using the sociodemographic form, Maslach Burnout Inventory (MBI), and Turnover Intention Questionnaire. Data were analyzed with SPSS and Amos software version 22 using independent *t*-test, ANOVA, and structural equation model.

**Results:**

The mean scores for burnout in emotional fatigue, depersonalization, and personal accomplishment dimensions were 25.38 ± 7.55, 9.47 ± 4.40, and 34.94 ± 7.80, respectively, moreover for the turnover intention, the score was 6.51 ± 3.17. The reduced personal accomplishment was identified as a positive predictor of turnover intention (*p* = 0.01). Work position and interest in attending the organization were significantly correlated with the turnover intention (*p* < 0.05).

**Conclusions:**

There is an immediate need to prepare nurses to cope better with the COVID-19 outbreak. Work-related stressors during the COVID-19 pandemic have led to an increase in nurses' burnout and turnover intention. Identifying and managing the factors related to professional burnout will make it possible to prevent the nurses' turnover intention in such critical situations.

## Introduction

In the twenty-first century, the coronavirus disease 2019 (COVID-19) pandemic and its negative consequences are a health threat to the people worldwide (1, 2). After a short time, COVID-19 has caused significant damage to public health while causing a financial and economic loss in many countries (3). Healthcare workers (HCWs), especially nurses worldwide, have played a significant role during disease outbreaks. Unpredicted stress exerted by the pandemic on every country's healthcare system has presented many difficulties for nurses (4). Additionally, the lack of personal protective equipment causes them to spread COVID-19 and distance from the workplace. Therefore, reducing the nursing staff increases the workload and extreme fatigue among other employees (5). In addition, healthcare providers are constantly dealing with the unpredictable sources of stress and situations that have many negative adverse on their physical and psychological health. These resources can include the nature of the job, high workload, high emotional load, the imbalance between demands and available resources, long working hours, long shifts, vague expectations, and weakness in supportive and effective management styles (6, 7). Viral threats, such as acute respiratory infections, also help exacerbate the health problems of nurses (8). Aprevious research had shown a variable level of nurses' intention to leave their profession across the globe. According to these studies, at the time of the outbreak of infectious diseases, such as severe acute respiratory syndrome (SARS), avian influenza (AV), and Middle East respiratory syndrome (MERS) (MERS-CoV), has shown that such outbreaks influence the interest of HCWs in their jobs (9). In addition, it affects attention, perception, and ability to make workplace decisions, productivity (8), dissatisfaction, reduced efficiency, burnout, turnover, burnout, and ultimately the tendency to leave the profession in nurses (10–12). Occupational burnout results from long-term exposure to certain job demand that a person is unable to bear (13). This syndrome is in the form of physical, mental, and emotional fatigue and a feeling of reduced personal success that leads to a variety of physical and mental illnesses, negative self-image, negative attitude toward the profession, lack of effective communication with the client, decreased patient safety, quality patient care, as well as turnover intention (14). The results of studies on burnout in nurses before and during the outbreak of coronavirus pandemic are reported to be moderate to high (15, 16). In Iran, the rate of stress and burnout is higher in nurses working in COVID-19 wards (13, 17). Turnover intention is one of the negative consequences of fatigue on HCWs. It is a common issue among nurses locally and internationally (18). In the last decade, the shortage of nurses has been a serious concern in most of the countries.

Intention to leave and subsequently leaving the job is one of the most important organizational factors that, if it occurs, can have devastating effects and financial burden, and high costs for the organization (19). Turnover intention means the departure of an organization's workforce over a certain period. Willingness to leave is a significant predictor of actual exit. It is also a cognitive stage that occurs before leaving the actual service and refers to a person's thought or mental decision about staying or leaving the job (20). Due to the heavy workload and stress, the rate of tendency to leave the nursing profession has the highest rank compared with other medical professions, and also the rate has varied from country to country, so that it has been reported in Asian countries 15 and 25%, respectively (21, 22), among western countries, such as the United States, 18% (23) and in Iran, 32.7%, respectively (24). However, the intention to leave during the COVID-19 outbreak was mentioned as one of the negative consequences and the reasons for it were anxiety, fear, and burnout of nurses (11, 12). According to previous studies, the high prevalence of psychological problems in COVID-19 has led to the tendency of employees to leave or reconsider their job choices or to help nurses exit (25–27). Therefore, the loss of experienced nurses has a negative impact on the provision and continuity of patient care services and may lead to more side effects, loss of nursing care, and patient mortality (28).

Given the widespread consequences of burnout and its impact on turnover intention in HCWs, particularly nurses, it is vital to understand and overcome this emerging problem (27). Because of an emerging infectious disease, such as COVID-19 can occur anywhere globally, health managers need to be aware of job stress, burnout, and its impact on employee propensity to leave. The results of previous studies have shown that positive organizational resources and work environment help reduce the tendency to leave the job. These resources have included providing opportunities for promotion and growth, increasing rewards, and emotional support for managers (29, 30).

Therefore, assessing the turnover intention of nurses is necessary to plan nurses' retention mechanisms in the Iranian context. This study is significant to add evidence for policy planners and program managers to improve such problems. Therefore, this study was conducted to investigate the dimensions of burnout and nurses' turnover intention who have experienced direct patient care in the first wave of COVID-19 in medical wards.

## Methods

### Design/Participant

This cross-sectional study is based on the structural equation modeling performed 6 months after the COVID-19 pandemic in the period from September to December 2020 in Iran. The study population consisted of 400 nurses (nurses, assistant nurses, and nursing students) working in the front line of two referral hospitals for patients with COVID-19 in Tehran. At the onset of the outbreak in early 2020, more than 10 wards for patients with COVID-19 were opened in these two referral hospitals, such as intensive care units (ICUs), internal medicine, emergency department, and day clinic and outpatient wards. The capacity of hospitalized patients was estimated at more than 200 patients per day.

Inclusion criteria were nurses and assistant nurses working in departments related to the patients with COVID-19, no physical or mental illness based on self-report, willingness to participate in the study, and completing the questionnaire.

### Procedure

In this study, due to the prevalence of the disease and the limitations related to the physical presence of researchers in medical centers, the questionnaires were converted into online versions, and its link was randomly shared for 200 nurses in nursing groups through social networks, such as WhatsApp, Telegram, or *via* email. Nurses formed these groups during the COVID-19 pandemic to meet the educational and scientific needs of treatment, care, and the latest guidelines issued by the Ministry of Health. After coordinating with the group administrators, the researchers sent a questionnaire link. The questionnaire was designed in Google Docs. The study samples were provided with explanations on the first page of the questionnaire, such as the title, purpose of the study, inclusion criteria, and ethical considerations.

### Data Collection

Data collection tools include 3 questionnaires: job and demographic information questionnaire, such as work position (nurse, assistant nurse, and student), age, gender, work experience, marital status, education level, satisfaction with income level, interest in attending the organization, experience in caring for patients with COVID-19, and describing the quality of sleep in the past month. The second questionnaire, Maslach burnout inventory-human service survey (MBIHSS), which is an internationally known, validated, self-report questionnaire for measuring frequency and severity of workplace burnout. It was first designed and used by Maslach et al. (1981) in the form of a Likert scale to assess the frequency and severity of the three dimensions of burnout (31). This questionnaire consists of 22 questions in the three dimensions of burnout, which include 8 questions related to emotional fatigue, 5 questions related to depersonalization, and 9 questions related to individual achievement (self-efficacy). The frequency of these emotions is from zero to 6 (never, several times a year, once a month, several times a month, once a week, several times a week, and every day). So that higher scores in the dimensions of emotional fatigue and depersonalization and lower scores in individual achievement indicate more burnout. The levels of emotional exhaustion (<17 low, 18–29 medium, 30 or higher, severe), the later levels of depersonalization (<5 low, 6–11 moderate, 12 and above, severe), and the levels of personal accomplishment [33 and less low, 34–39 moderate, 40 and more, severe (32)].

The Persian version of the questionnaire has been validated in Iran, and its Cronbach's alpha was between 0.86 and 0.96 (32, 33).

The third questionnaire, The Michigan Organizational Assessment Questionnaire, the tendency to leave of Cammann et al., has 3 questions and is based on a Likert scale from 1 to 5 (strongly agree to strongly disagree) and is in the range of 3–15. The average score was 9, score 3 indicates the lowest, and score 15 indicates the highest tendency to leave the service (34). Its Cronbach's alpha value in this study was 0.80. The Persian version of the questionnaire has been validated in Iran, and its Cronbach's alpha was 0.82 (35).

### Data Analysis

Data were analyzed with SPSS and AMOS statistical software version 16 using independent *t*-test, one-way analysis of variance (ANOVA), backward linear regression analysis, and structural equation modeling (**SEM**). The one-sample Kolmogorov–Smirnov test was performed to check the normality of data distribution, and the result showed normal data distribution (*p* > 0.05).

Bivariate Pearson's correlation coefficients and structural equation modeling were used to test the association between the dimensions of burnout and the nurses' turnover intention. The overall model fit was evaluated using P ratio, comparative fit index (CFI), Tucker–Lewis index (TLI), relative fit index (RFI), normal fit index (NFI), root mean square error of approximation (RMSEA), and relative chi square (CMIN/df).

### Ethical Consideration

The ethics committee has approved the present study of Baqiyatallah University of Medical Sciences with No. IR.BMSU.REC.1399.074. In this study, the voluntary and informed participation of the subjects, satisfying the respondents regarding the research by committing to not disclose their personal information in any way, and designing the questionnaires anonymously so as not to reveal the identity of individuals (maintaining confidentiality and anonymity) and obtaining permission from the Ethics Committee has been considered.

## Results

Findings from the analysis of 170 participants (85% response rate) showed that the mean age was 35.15 ± 10.12 years (range 20–62 years). The mean scores of burnout dimensions included emotional fatigue, depersonalization, and personal accomplishment were 40.38 ± 7.55, 9.47± 4.25, and 34.94 ± 7.80, respectively. Moreover, among nurses, 135 (79.4%) had a moderate and low, and only 35 (20.6%) had a high tendency to leave the service. The mean score of turnover intention was 6.51 ± 3.17. There was no significant relationship between gender, marital status, the level of education, care of patient with COVID-19, clinical work experience, satisfaction with income level, and sleep quality with nurses' turnover intention (*p* > 0.05). However, the mean scores of job type and interest in the organization had a positive relationship with nurses' turnover intention. Least significant difference (LSD) *post-hoc* test showed that assistant nurses were significantly more likely to exit than nurses (*p* = 0.02) and students (*p* = 0.009). Furthermore, the mean score of turnover intention among nurses who were less interested in the organization was significantly higher than the other two groups (*p* < 0.001) (Table 1). Multiple regression analysis demonstrated that the work position and interest in attending the organization were significantly associated with the Turnover Intention score.

**Table 1:** Socio-demographic information of participants.

**Variable**		**Number**	**Percentage**	**Turnover Intention score** **Mean ±SD**	***P*-value**
Gender	Male	102	60	6.39 ± 3.12	0.52
	Female	68	40	6.70 ± 3.26	
Marital status	Single	52	30.6	6.67 ± 3.30	0.67
	Married	118	69.4	6.44 ± 3.12	
Educational status	Associate	53	31.2	6.47 ± 3.27	0.87
	Bachelor	81	47.6	6.44 ± 3.07	
	Master	24	14.1	7.00 ± 3.10	
	Ph.D.	12	7.1	6.25 ± 3.79	
Experience in caring of a patient with Covid-19	Yes	101	77.1	5.81 ± 3.13	0.52
	No	30	22.9	5.40 ± 3.02	
Work position	Nurse	119	70.4	6.40 ± 3.17	0.02
	Nurse assistant	23	13.6	8.04 ± 2.65	
	Student	27	16	5.70 ± 3.29	
Work experience	<6 year	65	38.7	6.44 ± 3.06	0.24
	6–10 year	28	16.7	6.78 ± 3.57	
	11–15 year	19	11.3	6.63 ± 3.02	
	16–20 year	18	10.7	5.00 ± 2.42	
	>20 years	38	22.6	7.07 ± 3.39	
Income satisfaction	Low	16	9.4	7.43 ± 2.87	0.23
	Moderate	92	54.1	6.17 ± 3.00	
	High	62	36.5	6.79 ± 3.46	
Interest in attending the organization	Low	21	16.2	8.85 ± 4.26	>0.001
	Moderate	57	43.8	5.85 ± 2.74	
	High	52	40	4.30 ± 1.73	
Sleep quality	Very bad	20	15.3	6.50 ± 3.56	0.07
	Fairly bad	40	30.5	6.47 ± 3.68	
	Fairly good	54	41.2	5.18 ± 2.65	
	Very good	17	13	4.70 ± 1.57	

The findings of correlation coefficients between the different dimensions of burnout and the score of intention to leave showed that with increasing the three dimensions of burnout, scores related to the tendency to leave increases, but the relationship is not significant. As the individual's achievement decreases, the emotional fatigue and depersonalization dimensions' scores increase (Tables 2, 3).

**Table 2:** Pearson correlation coefficients between different dimensions of burnout with each other and turnover intention.

	**Emotional fatigue**	**Depersonalization**	**Personal accomplishment**	**Turnover intention**
Emotional fatigue	1	–	–	–
Depersonalization	0.50 (*P* < 0.001)	1	–	–
Personal accomplishment	−0.13 (*P* = 0.09)	−0.28 (*P* < 0.001)	1	–
Turnover intention	0.12 (*P* = 0.12)	0.15 (*P* = 0.05)	−0.14 (*P* = 0.06)	1

**Table 3:** Regression coefficients related to the association between burnout dimensions and turnover intention.

	**Standardized regression coefficient**	**Regression coefficient**	**S.E**	**Test statistics**	***p*-value**
Emotional fatigue	0.072	0.078	0.15	0.52	0.6
Depersonalization	0.201	0.299	0.22	1.32	0.18
Personal accomplishment	−0.263	−0.265	0.10	−2.58	0.01

**Figure 1:**
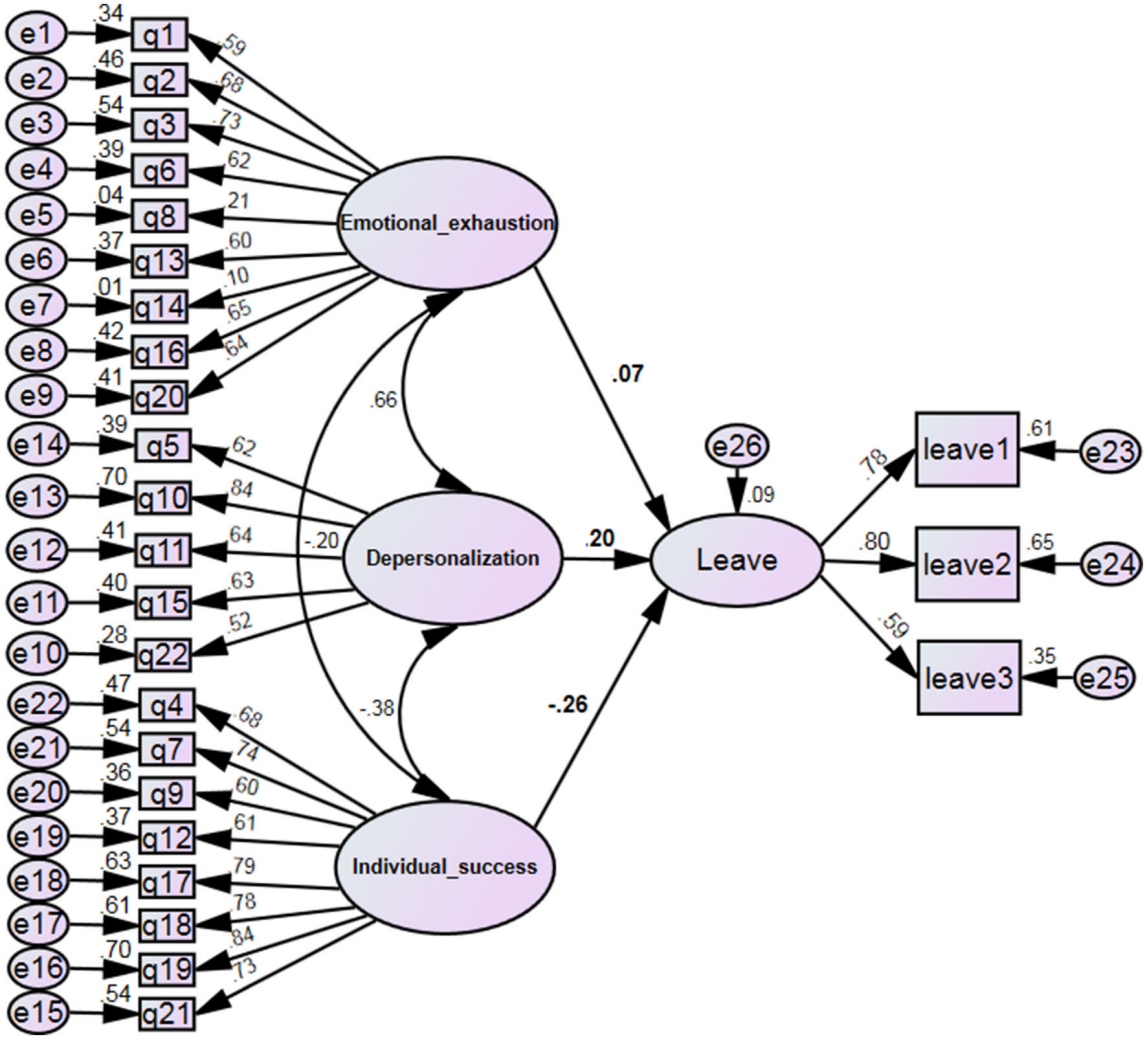
Structural model of research with standard coefficients.

The results of the structural equation showed that although the effect of emotional fatigue and depersonalization dimensions on intention to leave was not statistically significant (*p* > 0.05), there was a statistically significant relationship between the personal accomplishment component and job leaving. Thus, it can be said that by increasing one unit in the individual accomplishment score, the average score of turnover intention will be 0.26 less (Table 4 and Figure 1).

**Table 4:** Model fit indices in examining the relationship between the dimensions of burnout and turnover intention.

**PRATIO**	**CFI**	**TLI**	**IFI**	**RFI**	**NFI**	**RMSEA**	**CMIN/DF**
0.9	0.85	0.83	0.85	0.7	0.73	0.07	1.92

The model fit indices are given in Table 4. The calculated values indicate that the model's slight negligence fit is acceptable.

## Discussion

The present study aimed to assess the dimensions of professional burnout and turnover intention among nurses working in hospitals during the COVID-19 pandemic. The present study results revealed that nurses suffered moderate burnout during the coronavirus crisis. However, since only 6 months passed from the outbreak of COVID-19 until the present study, the rate of burnout was significant and might increase if not prevented. The spread of infectious diseases over the past two decades has been a severe threat to the health system worldwide. Healthcare providers are under a great deal of physical and psychological pressure to care for many potential infectious victims. Therefore, burnout is not a new phenomenon. In line with the present study, the results of other studies showed that the majority of nurses working in the front line of COVID-19 had experienced 19 degrees of mild to high levels of burnout (4, 21). In addition, the present results showed that the prevalence of burnout in nurses, who were at the forefront of COVID-19, was much higher than the mean score of previous studies. Thus, immediate significant preventative considerations (36, 37) focused on the study objective, nurses' intention to live in the current area at the time of COVID-19 was low, in line with the results of other studies in Iran (19). Although the assessment tool in the present study was different from the above two studies, the tendency to leave among nurses was reported in the medium and low range. This finding is consistent with the Philippines study (11). However, in another study in Iran, the tendency to leave service during the COVID-19 epidemic was reported to be higher (38).

Evidence suggests that the tendency to leave varies among nurses in different communities, depending on the severity of the viral disease outbreak. These differences may be attributed to the multiple definitions of the phenomenon of intent to leave due to differences in the research setting and even the duration of the COVID-19 outbreak. The direct contact of health workers with patients and observing their COVID-19 can increase the rate of intention to leave. According to previous studies, job stress, anxiety, and nurses' fear of coronavirus disease have increased the intention to leave among them (11). In addition, there is a positive relationship between the tendency to leave with job stress which predicts the tendency to leave among nurses (19). Moreover, according to the study results, with the increase in the dimensions of burnout, employees were more inclined to leave. So that burnout in the dimension of personal accomplishment had the greatest role in leaving intentions among nurses. In line with the present study, the highest prevalence of burnout has been reported about the decreased personal accomplishment (36, 39). The feelings of decreased personal accomplishment are described as decreased production capacity and individual ability, low morale, and inability to cope with problems (14).

Conversely, the feeling of personal success increases the job satisfaction, reduces the feeling of failure and disability, and consequently increases productivity (40). Additionally, a sense of personal success, desire to continue working, and professional presence is created among nurses when they see the improvement of patients due to their care efforts, which significantly reduces the work stress of nurses (38). Other reasons may have been the nurses' lack of previous exposure or experience in caring for patients with COVID-19 or similar pandemics, such as SARS or MERS, inadequate knowledge, frequent changes in the disease process, changes in guidelines have caused frequent worries, loss of confidence, feelings of inefficiency, and also the tendency of nurses to leave the service. The study results related no significant correlation between gender and the tendency to leave. These results were in line with other studies (22, 24, 38).

Conversely, the study conducted by Mirzaei (19) was significantly correlated with the variable of gender with a higher turnover intention. This difference may be attributed to the cultural context and setting of the study. It can also be said that this study was conducted in the first wave of COVID-19 in Iran, and this has probably affected the rate of intention to leave male and female nurses equally.

The results of the present study revealed that the mean turnover intention among nurses was not significantly correlated with the variables of marital status, level of education, experience in caring of a patient with COVID-19, clinical work experience, income satisfaction, and sleep quality, which was in line with other studies (38). A study showed that young and employed nurses in the private sector are more likely to leave (41). On the other hand, in another study, married and highly experienced nurses were more likely to leave due to fear of infection, burnout, and increased risk perception (27). The reason for such difference might be the tendency of nurses to leave is influenced by their care of patients and has less to do with their educational status. On the other hand, perhaps the nursing profession's critical conditions and altruistic nature have caused different degrees of non-difference of nurses. In the present study, the tendency to leave was not significantly associated with income satisfaction. Conversely, another study, low salaries reduced the quality of care and motivated nurses and increased the tendency to leave (42). Perhaps the organizational culture as well as the moral commitment to care in the times of crisis has been very prominent among healthcare providers. In addition, according to previous pieces of evidence and experiences, the commitment to work, love, and self-sacrifice of Iranian nurses in the current crisis is beyond material issues.

The study results showed that nursing assistants had more turnover intention than nurses. In the health system in Iran, nursing assistants are under more work pressure and stress due to their duties, job expectations, and type of care delivery. At the COVID-19 outbreak, they were more likely to be infected due to their high workload.

The present study results also showed that turnover intention had a positive and significant correlation with job satisfaction. These results align with those of the study conducted by Varasteh et al. (18). Job motivation, job satisfaction, and perceived organizational support are the predictors of nurses' tendency to leave their jobs (19). Organizations that provide more employee support are more likely to reduce stress and ultimately increase employee retention. Since managers in this organization can apply effective policies and methods to protect the human resources before employees leave. The present study in 6 months after the first COVID-19 wave in Iran and the study of various factors on the tendency of retention in HCWs can be an innovative aspect. Therefore, using the results of this study to assess the situation of employees in the current crisis and other various health crises in the future can be useful for planning the managers and policy makers of the health system.

## Limitations

Small sample size is one of the limitation of study, so studies with higher sample sizes may offer different results. The use of self-report questionnaires may have created response biases. This study was performed in two COVID-19 reference hospitals in Tehran. Therefore, future studies can examine other hospitals according to the structure, culture, and organizational climate. The cross-sectional study design makes it difficult to explain the causal relationship between risk factors and turnover intention. The findings of this study may not be generalizable to the nurses' population in Iran as a whole. This study was conducted 6 months after the first wave of COVID-19. So, future research should be considered to assess the turnover intention and the level of burnout at different times of the COVID-19 epidemic. Finally, questionnaires were sent and completed online due to the limited access to research samples. So, we could not comply fully with our sampling schedule and plan. In future research, face-to-face questionnaires and interviews, observation of behavior in the workplace, and peer reporting are recommended.

## Conclusion

Work-related stressors during the COVID-19 pandemic have led to an increase in the nurses' burnout and turnover intention. The present study results showed that nurses experience the moderate levels of burnout during the COVID-19 pandemic, while several sociodemographic and occupational factors affect this burnout and turnover intention. Reduced personal accomplishment is the most predictor for turnover intention. Thus, these factors should be identified and managed to prevent turnover intention in such critical situations. Most importantly, coping strategies to reduce stress during the outbreaks of infectious disease through the support of co-workers, caregivers, and supervisors should be actively used by nurses to reduce their turnover rates. To reduce the nurses' turnover intention and improve their mental health, healthcare managers and policymakers need to plan to prepare healthcare systems, individuals, and nurses for a better response to the COVID-19 outbreak.

## Data Availability Statement

The raw data supporting the conclusions of this article will be made available by the authors, without undue reservation.

## Ethics Statement

The present study was approved by the Ethics Committee of Baqiyatallah University of Medical Sciences, Tehran, Iran, with code IR.BMSU.REC.1399.074. The patients/participants provided their written informed consent to participate in this study.

## Author Contributions

LK: data collection and writing—review and editing. MR: methodology, data analyze, and writing—review and editing. AP: conceptualization, methodology, writing—review and editing, and supervision. All authors contributed to the article and approved the submitted version.

## Conflict of Interest

The authors declare that the research was conducted in the absence of any commercial or financial relationships that could be construed as a potential conflict of interest.

## Publisher's Note

All claims expressed in this article are solely those of the authors and do not necessarily represent those of their affiliated organizations, or those of the publisher, the editors and the reviewers. Any product that may be evaluated in this article, or claim that may be made by its manufacturer, is not guaranteed or endorsed by the publisher.
